# Toward a Model for Activation of Orai Channel

**DOI:** 10.1016/j.isci.2019.05.041

**Published:** 2019-06-01

**Authors:** Hao Dong, Yiming Zhang, Ruiheng Song, Jingjie Xu, Yigao Yuan, Jindou Liu, Jia Li, Sisi Zheng, Tiantian Liu, Benzhuo Lu, Youjun Wang, Michael L. Klein

**Affiliations:** 1 Kuang Yaming Honors School, Nanjing University, Nanjing 210023, People's Republic of China; 2 Beijing Key Laboratory of Gene Resource and Molecular Development, College of Life Sciences, Beijing Normal University, Beijing 100875, People's Republic of China; 3 State Key Laboratory of Scientific and Engineering Computing, National Center for Mathematics and Interdisciplinary Sciences, Academy of Mathematics and Systems Science, Chinese Academy of Sciences, Beijing 100190, People's Republic of China; 4 Institute for Computational Molecular Science, Temple University, Philadelphia, PA 19122, USA; 5 CAEP Software Center for High Performance Numerical Simulation, Beijing 100088, People's Republic of China; 6 Institute for Brain Sciences, Nanjing University, Nanjing 210023, People's Republic of China

**Keywords:** Biological Sciences, Molecular Biology, Structural Biology

## Abstract

Store-operated calcium release-activated calcium (CRAC) channels mediate a variety of cellular signaling functions. The CRAC channel pore-forming protein, Orai1, is a hexamer arranged with 3-fold symmetry. Despite its importance in moving Ca^2+^ ions into cells, a detailed mechanistic understanding of Orai1 activation is lacking. Herein, a working model is proposed for the putative open state of Orai from *Drosophila melanogaster* (dOrai), which involves a “twist-to-open” gating mechanism. The proposed model is supported by energetic, structural, and experimental evidence. Fluorescent imaging demonstrates that each subunit on the intracellular side of the pore is inherently strongly cross-linked, which is important for coupling to STIM1, the pore activator, and graded activation of the Orai1 channel. The proposed model thus paves the way for understanding key aspects of calcium signaling at a molecular level.

## Highlights

• Mechanical coupling within the calcium channel pore is critical for its activation
• Molecular modeling could disclose gating mechanism of ion channels at atomic level
• The predicted open-state structure of the pore was further confirmed by experiments

## Graphical Abstract

**Figure:**
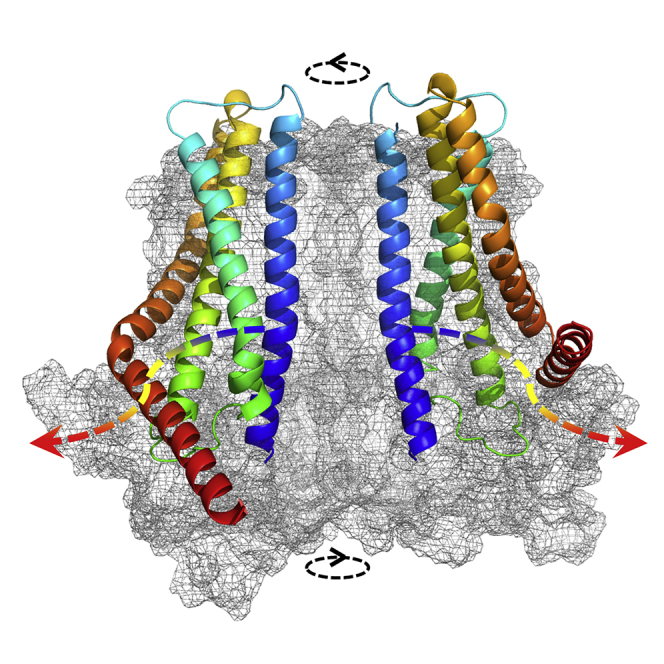

## Introduction

Calcium ions (Ca^2+^) play an important role in almost every aspect of cellular life (Clapham, 2007). The Ca^2+^ release-activated Ca^2+^ (CRAC) channel is one of the major pathways for Ca^2+^ communication between extracellular and intracellular environments. The CRAC channel, which is operative in response to Ca^2+^ depletion in the endoplasmic reticulum (ER) (Ali et al., 2016, Prakriya and Lewis, 2015, Hogan et al., 2010), is characterized by its extraordinarily high Ca^2+^ selectivity over monovalent ions (P_Ca_/P_Na_>1,000) and low unitary conductance (Prakriya, 2009). The CRAC channel consists of two key components, the pore-forming protein Orai1 located at the plasma membrane (PM) (Vig et al., 2006, Prakriya et al., 2006, Feske et al., 2006) and the ER-resident Ca^2+^ sensor STIM1 (Roos et al., 2005, Liou et al., 2005). The depletion of ER Ca^2+^ triggers the oligomerization and activation of STIM1 as well as its translocation from the ER membrane to the ER-PM junction, where it is in close contact with Orai1 and is thus able to activate the channel (Feske et al., 2012). Despite the importance of CRAC channels, and ongoing efforts to explore their functioning, the gating mechanism, and especially how the pore structure rearranges upon activation, remains elusive (Shim et al., 2015).

**Figure 1:**
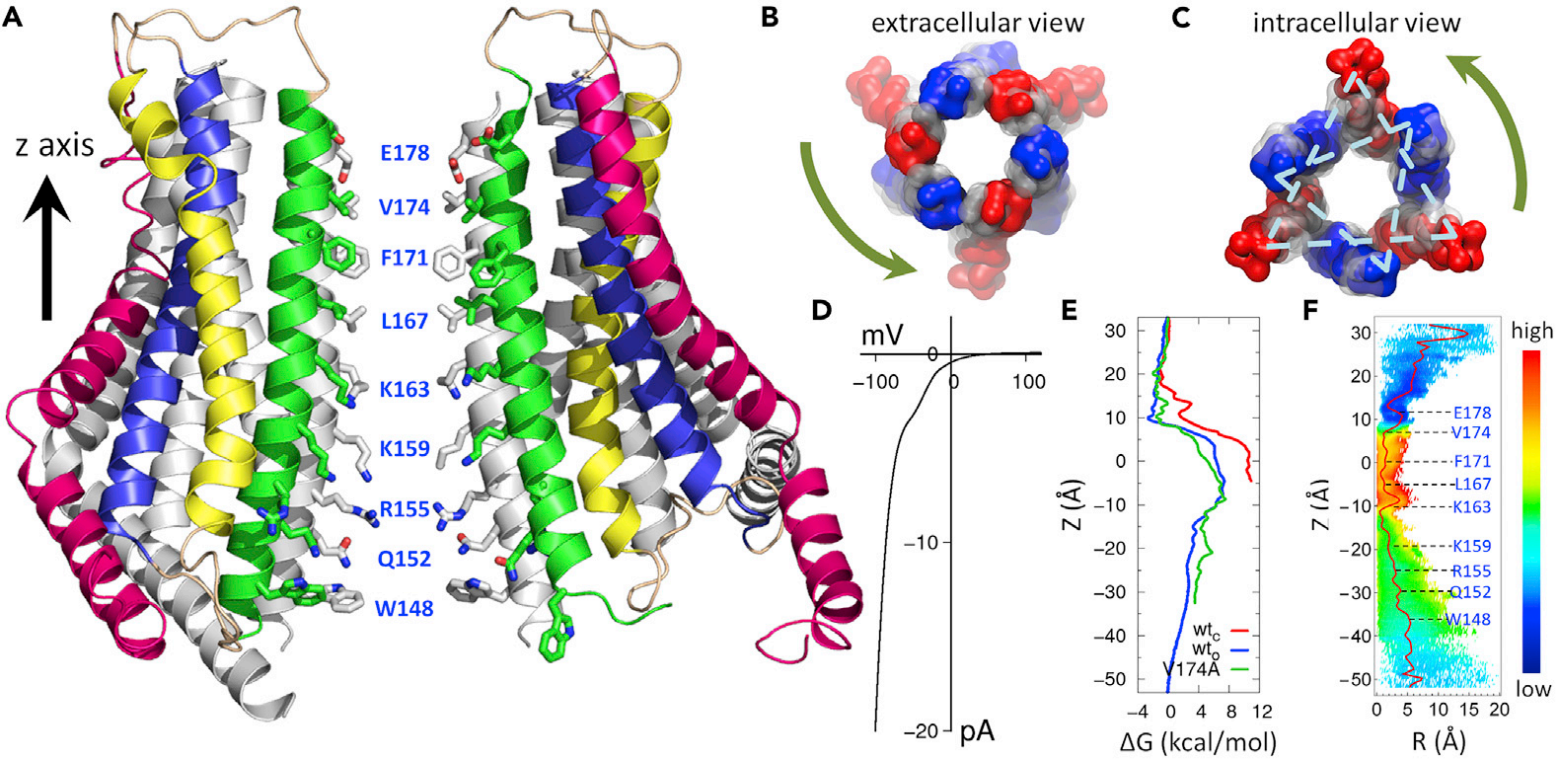
The Putative Open-State Structure of dOrai and the “Twist-to-Open” Gating Motion (A) A representative open-state structure of the wild-type dOrai pore (abbreviated as wt_o_, where TM1–TM4 are in green, yellow, blue, and red, respectively) aligned with its X-ray structure in the closed state (abbreviated as wt_c_, which is shown in silver, PDB entry: 4HKR). Key residues lining the pore are shown in stick mode. (B and C) Superposition between wt_c_ (in gray) and wt_o_ (in red and blue) pores, where only TM1 helices are shown in space-filling mode. Views from the extracellular (B) and intracellular (C) sides of the pore highlight the different directions of rotation during gating (shown by arrow). The dashed line connects Cα atoms of Q152 on alternate chains, representing different motions of neighboring TM1 at the N-terminal side. (D) The calculated I-V relationship of the wt_o_ structure. (E and F) Calculated potential of mean force (PMF) for cation permeation through the pore. (E) The one-dimensional PMFs of wt_o_ (in blue), wt_c_ (in red), and the V174A mutant (in green), showing that the open state is energetically favorable for ion flux. (F) The two-dimensional profile of the wt_o_ further suggests a zigzag pathway for ion permeation along the pore. The x axis shows the distance away from the pore axis; the y axis is along the pore axis.

The structure of Orai from *Drosophila melanogaster* (dOrai) at 3.35 Å resolution has been reported with a closed state of the pore formed by six subunits adopting a 3-fold rotational symmetry (Hou et al., 2012). In each subunit, there are four transmembrane helices (TM1–TM4), with a C-terminal helical cytosolic extension of TM4 (TM4-ext) (Figure 1A). Experimental observations have identified multiple sites or domains as being crucial for Orai1 activation, and intra-subunit interactions are also known to be important (Yamashita et al., 2017, Yeung et al., 2018, Palty et al., 2015, Fahrner et al., 2017, Zhou et al., 2016, Dynes et al., 2016, Gudlur et al., 2014). Both dilation of the pore (Hou et al., 2012, Zhang et al., 2011) and rotation of TM1 (Yamashita et al., 2017, Yeung et al., 2018, Gudlur et al., 2014) have been proposed to be critical for gating.

The study of mutants provides an alternative means for exploring gating especially those that are constitutively conducting in the absence of STIM1 (Palty et al., 2015, Zhang et al., 2011, Frischauf et al., 2017, Mcnally et al., 2012). Zhou et al. applied multiple mutations in the TM4/TM4-ext region and identified the unexpected correlation between the central pore and peripheral segments for Orai activation (Zhou et al., 2016). Yamashita et al. focused on the V102/F99 double mutants and found that physical occlusion of the hydrophobic residues is critical for gating, and gating was achieved by rotating pore-forming helices (Yamashita et al., 2017). This finding is consistent with our results obtained from molecular modeling that pore hydration has profound effects on its gating (Dong et al., 2013, Dong et al., 2014). Substitution of residue H134 in hOrai1 (equivalent to H206 in dOrai) with alanine leads to a constitutively open pore with almost no impaired Ca^2+^ selectivity, suggesting increased pore size and higher flexibility of the basic region (Yeung et al., 2018, Frischauf et al., 2017). Very recently, a published X-ray crystal structure of the H206A mutant of dOrai at 6.7 Å resolution shows pore widening and a significant conformational change of the TM4-ext, although rotation of the TM helices is not evident (Hou et al., 2018). In addition, in Orai from *Caenorhabditis elegans*, the intracellular loop was found to be involved in gating, which was not proposed for mammalian Orai (Kim et al., 2018). Gating of the channel was also affected by some cofactors such as lipid composition. For example, the absence of cholesterol binding at the N terminus of Orai leads to enhanced Ca^2+^ influx (Derler et al., 2016). These observations provided different insights into the activation of Orai. However, the relevance of the abovementioned information for Orai gating under physiological conditions is unclear, as the conformational changes associated with wild-type Orai1 activation remain ill defined, and no working model is available that can reconcile all the observations.

This situation motivated the use of computer modeling to identify possible missing information concerning channel gating. If the inherent dynamics of the pore protein plays a dominant role in determining the structural changes induced by STIM binding, then a normal mode analysis (NMA) provides a robust computational tool that is able to reveal a relationship between structure and dynamic behavior (Bahar et al., 2009). Indeed, NMA combined with molecular dynamics (MD) simulations has been employed to successfully characterize the coupled motion between the ligand-binding ectodomain and the TM domain of trimeric ATP-gated P2X4 receptor during activation (Du et al., 2012), as well as the gating process of other membrane proteins such as KcsA (Shen et al., 2002), the nicotinic acetylcholine receptor (nAChR) (Taly et al., 2005), and the acid-sensing ion channel 1 (Yang et al., 2009), etc.

In the present work, a combined NMA and MD protocol was employed to explore the gating of the wild-type Orai pore and thus infer a possible open-state structure (Figure 1A). The putative open-state structure was supported by energetic, structural, and additional experimental evidence. Specifically, the free energy profile for cation permeation via the proposed open-state structure is found to have a major barrier height as low as ∼8 kcal/mol. And the calculated current-voltage (I-V) curve reproduces the characteristic inward rectification with a reversal potential of ∼64 mV, suggesting high Ca^2+^ selectivity. Based on the present putative open-state model, two salt bridges, R155-E221 and K157-E245 in dOrai (equivalent to R83-E149 and K85-E173 in hOrai1, respectively), which were identified to be critical for gating by transmitting conformational change, were further tested with protein engineering, imaging, and whole-cell patch clamp measurements. The results of all these studies support the notion that coupling between TM1 and TM3 is crucial for STIM1-mediated cross-linking and graded activation of Orai channels.

The experimental data from the present work provides persuasive evidence in favor of the proposed “twist-to-open” gating mechanism for the wild-type pore under physiological conditions, which should thus serve as a reliable model for understanding CRAC channel function.

## Results

### The “Twist-to-Open” Gating Motion

Based on the X-ray structure of dOrai in the closed state (Hou et al., 2012), we explored a possible open-state structure derived from NMA and then further relaxed the structure with sub-microsecond MD simulations. The putative channel gating mode of the wild-type dOrai was selected based on the following known experimental evidence for its activation: first, a minimal perturbation at residue E178 is introduced to maintain the rigidity of the selectivity filter (Gudlur et al., 2014); second, constraints at the major gate of the channel, the hydrophobic region including V174 and F171 (Yamashita et al., 2017, Mcnally et al., 2012), as well as the first positively charged residue K163 (Dong et al., 2013, Dong et al., 2014), are partially released; next, motions of the cytosolic side of TM helices are coupled (Kim et al., 2018, Zhou et al., 2018); then, the coiled-coil motif formed by neighboring TM4-exts are broken (likely facilitating STIM binding) (Hou et al., 2018, Tirado-Lee et al., 2015, Stathopulos et al., 2013); and finally, both the local gating motion and the global collective motion of the entire protein are assumed to be symmetric (Dai and Zhou, 2014). These computations identified a “twist-to-open” gating motion. Notably, clustering of the MD simulations trajectory based on the wild-type dOrai at the closed-state structure revealed similar motion patterns (Figure S1).

In general, the channel twisted around the central pore upon gating, and different regions show quite distinct gating motions. To be specific, the extracellular side of the protein (including the six TM1 helix bundles) has a collective motion that undergoes counterclockwise rotation, with a subtle expansion of the pore-forming TM1 bundle (Figure 1B); the intracellular side of this bundle, on the other hand, has two independent types of motion: the N-terminal section of TM1 on three alternate chains moves outward (red in Figure 1C), whereas the other three chains undergo clockwise rotation (blue in Figure 1C).

The twist motion of the pore is characterized by the reorientation of pore-lining residues. Starting from the closed-state structure (Hou et al., 2012), the TM1 helices in the putative open-state structure experience a counterclockwise rotation of 14° at residue F171, and 12° at residue L167, but clockwise rotation of −58° at R155. The inner portion of the pore is dilated, as the interchain distances between Cα atoms on residue Q152 at the N-terminal section of TM1 are 22.3 (±2.3) Å on three alternate chains and 18.4 (±2.1) Å on the other three chains (Figure 1C), whereas the distance in the closed-state structure is 16.2 Å. Presumably, the energy needed to twist the pore and open the hydrophobic gate mainly comes from the STIM-binding event.

During the pore-breathing motion, the N-terminal sides of TM1 helices undergo two different conformational changes: clockwise rotation and outward bending (Figure 1C). This phenomenon is likely caused by two alternating conformations of TM4-ext on neighboring subunits, which are tightly packed together in the closed state (Hou et al., 2012) and get unpacked in the putative open state. Therefore the conformational change of the pore is transmitted through the coupling of TM helices (as further described below). Hence gating of the pore is governed by motions of the entire channel and involves its collective dynamics.

The observed channel motions lead to the release of the restrained pore without loosening of the overall structural integrity. The central pore was found to be more hydrated and pore waters were less ordered (Figure S2), a key characteristic for channel opening (Dong et al., 2013, Dong et al., 2014).

### Structure Validation

To evaluate the reliability of the aforementioned putative open-state structure, new computational and experimental evidences, along with data already reported in literature were employed, as described below.

#### I-V Characteristics of the Pore

Experimentally, the I-V relationship obtained from patch clamp recordings show a typical inward rectification with a reversal potential of ∼80 mV, which is a signature of the high Ca^2+^ selectivity of the CRAC channel (Mcnally et al., 2012).

The Poisson-Nernst-Planck (PNP) model is an approximate continuum model, which can capture certain macroscopic properties, such as the I-V characteristics. Here, the solvent continuum model combined with extensive MD simulations for conformational sampling was employed to characterize the ion diffusion profiles of the central pore of the putative open construct.

The calculated reversal potential for the putative open-state structure is 64 mV, which is close to the experimental value, thus suggesting support for the putative activated state structure with high selectivity for Ca^2+^ under physiological conditions. More importantly, the calculated I-V curve reproduced the inward rectification of the pore (Figure 1D), which could be attributed to the asymmetric distribution of charged residues at the entrance of the pore (Figure 1A).

#### Energetic Evidence for Pore Opening: Cation Permeation Free Energy Profile

The CRAC channel can conduct Na^+^ ions in the absence of divalent ions. As in previous work (Dong et al., 2013, Dong et al., 2014), permeation of Na^+^ through the pore was studied in this work, rather than Ca^2+^, mainly because the available parameters for the commonly employed CHARMM36 force field overestimate the binding affinities between Ca^2+^ and the protein by more than 80 kcal/mol (Li et al., 2015), a situation likely to result in a very biased computed permeation behavior for Ca^2+^. Given the very low conductance of hOrai1 channel under physiological condition, ∼700 fS for Na^+^ in the divalent-free environment (Prakriya and Lewis, 2006) (equivalent to ∼2 μs per permeation event), it is difficult to deduce the conductance value of the pore directly from conventional MD simulations. Consequently, the potential of mean force (PMF) for Na^+^ permeation through the putative open-state structure of the wild-type pore was calculated to confirm its opening, which is a well-established protocol used in previous computational works to explore channel conductance (Dong et al., 2013, Dong et al., 2014).

The PMF for Na^+^ permeation has a major free energy barrier height of ∼8 kcal/mol, 3 kcal/mol lower than the one for the closed state, showing a favorable pathway for ion permeation through the central pore (Figure 1E). The region limiting ion flux is relatively broad, which is formed by the hydrophobic region (V174, F171, and L167) together with the residue K163 in the basic region. Interestingly, the overall permeation pathway in the putative open state of dOrai resembles that in the V174A mutant, probably because both constructs release the physical constraint for ion permeation in a similar way: the V174A mutant leaves more space for the pore with its smaller side chain (Dong et al., 2013), and the putative open-state construct rotates its bulky hydrophobic side chain away from the central pore.

Rather than going straight through the lumen of the pore, cation motion follows a zigzag pathway (Figure 1F), which is likely due to the less well-coordinated geometry of an ion in the relatively narrow pore. Consequently, pore-lining residues readily trap the permeating cation. This finding partially explains the low unitary conductance of the Orai channel under physiological conditions.

#### Structural Evidence for Pore Opening: The R155-E221 Salt Bridge

**Figure 2:**
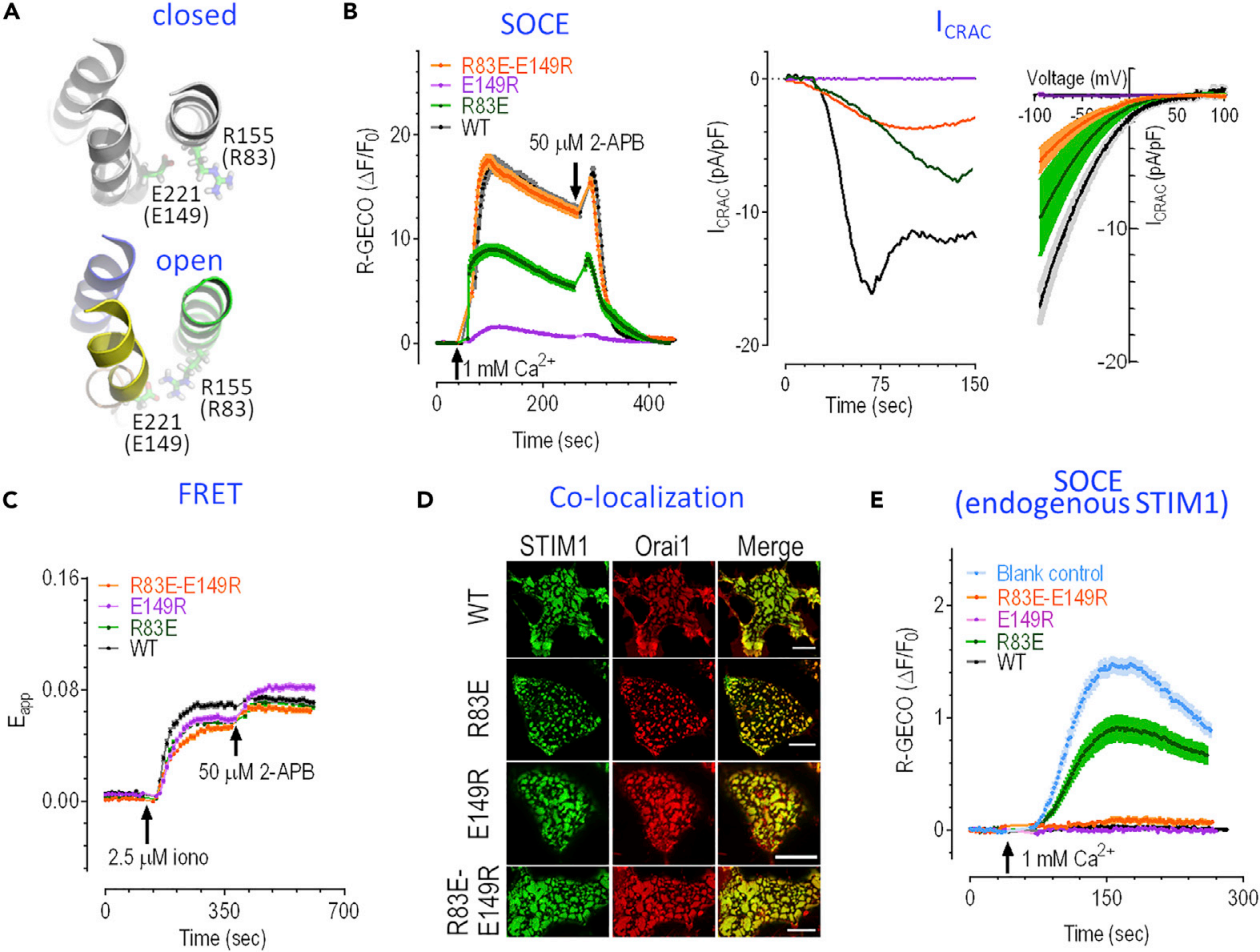
The Salt Bridge R155-E221 in dOrai Is Critical for the coupling between N and C Termini of the Pore (A) Gating motion involves the clockwise rotation of the N terminal of TM1, leading to the formation of the R155-E221 interaction to enhance coupling between the central pore and peripheral helices. (B–D) HEK STIM1-YFP cells transiently expressing wild-type CFP-Orai1 or corresponding mutants with similar CFP fluorescence levels. (B) Typical SOCE responses (left) and whole-cell current responses (middle, typical current traces measured at −100mV; right, mean I-V relationships measured at the peak of time traces) (n ≥ 5 for each condition). Mean SOCE (R-GECO [ΔF/F_0_]: wild-type [WT] = 13.90 ± 0.55 [n = 68], R83E = 7.48 ± 0.42 [n = 59], E149R = 1.00 ± 0.08 [n = 68], R83E-E149R = 13.81 ± 0.36 [n = 68]). Only SOCE responses from single mutants are significantly lower than those of WT (^∗∗∗^, t test). CRAC responses from all mutants are significantly lower than those from WT (^∗∗^, t test). (C) FRET signals between STIM1 and Orai1 before and after store depletion induced by 2.5 μM ionomycin. Ionomycin-induced peak ΔE_app_: 0.058 ± 0.002 (n = 74) for WT, 0.046 ± 0.001 (n = 78) for R83E, 0.048 ± 0.002 (n = 75) for E149R, 0.040 ± 0.001(n = 76) for R83E-E149R; all are significantly lower than that of WT (^∗∗∗^, t test). (D) Typical confocal images showing the co-localization of STIM1 and Orai1 after store depletion (please see Figure S5B for statistics). (E) The SOCE responses of HEK WT cells transiently expressing WT CFP-Orai1 or its corresponding mutants. Mean SOCE: WT = 0.03 ± 0.01 (n = 64), blank control = 1.05 ± 0.04 (n = 81), R83E = 0.63 ± 0.07 (n = 59), E149R = 0.00 ± 0.01, (n = 59), R83E-E149R = 0.06 ± 0.01 (n = 62). SOCE responses from R83E mutant and blank control are significantly bigger than that of WT (^∗∗∗^, t test). For (B), (D), and (E) before recording, cells were bathed in 0Ca^2+^ solution containing 1 μM TG (thapsigargin, a SERCA pump blocker) for 10 min to deplete ER Ca^2+^ store. 1 μM TG was present throughout the recordings. For current measurements, ER Ca^2+^ stores were passively depleted by 20 mM BAPTA (1,2-bis(o-aminophenoxy)ethane-N,N,N′,N′-tetraacetic acid, a potent Ca^2+^ chelator) included in pipette solution. At least three independent repeats were carried out for each experiment.

In the closed-state X-ray structure (Hou et al., 2012), the conserved residue R155 on the N-terminal segment in dOrai (equivalent to R83 in hOrai1) is projecting into the central pore. Although loop2, connecting helices TM2 and TM3, is missing in that structure (Hou et al., 2012), residue E221 on loop 2 (equivalent to E149 in hOrai1) cannot form an interaction with R155 in the closed state of the pore, as suggested by computer simulations (Dong et al., 2013). In contrast, the present putative open-state model suggests that the gating motion involves the clockwise reorientation of R155, which initially faced away from the pore and formed a salt bridge with E221 (Figure 2A). This observation prompted an experimental investigation of the impact of double mutations on hOrai1-R83X-E149X (X is either original or charge reversal residue).

When transiently expressed in HEK STIM1 stable cells, the expression level of these mutants were similar to that of WT Orai1 as indicated by their CFP fluorescence, and cellular distribution of these single mutants were similar to that of wild-type Orai1 (Figure S6). In contrast, both hOrai1-R83E and hOrai1-E149R show decreased Ca^2+^ influx after store depletion (left panel in Figure 2B), which based on our putative open-state structure could be attributed to the elimination of the R83-E149 salt bridge in hOrai1. A similar result was reported for the hOrai1-E149A mutant (Srikanth et al., 2010). In contrast, the hOrai1-R83E-E149R double mutation fully restored the diminished Ca^2+^ influx caused by single mutations, with a similar amplitude to that of the wild-type hOrai1 (left panel in Figure 2B) (Wu et al., 2013). At high concentration (50 μM), 2-Aminoethoxydiphenylborate (2-APB) would transiently potentiate store-operated Ca^2+^ entry (SOCE), and then diminish SOCE quickly, whereas it only increases Förster resonance energy transfer (FRET) signals between STIM1 and Orai1 (Navarro-Borelly et al., 2008). Such classical 2-APB-induced effects on Ca^2+^ influx and FRET responses in STIM1 cells expressing Orai1-R83E-E149R were observed (Figures 2B and 2C). When examined with whole-cell current measurements, the R83E-E149R double mutation also partially rescued the diminished current mediated by Orai1-E149R, with an inwardly rectifying I-V relationship, which is a signature for I_CRAC_ (right two panels in Figure 2C). It should be noted that even though hOrai1-R83E-E149R still had some minor defects in coupling with STIM1 (Figure 2C), indicating that the R83-E149 salt bridge is not essential for STIM1-Orai1 coupling, it may have an important role in intra-Orai1 conformational changes that govern Orai1 activation after its binding with STIM1. Nevertheless, it could clearly co-localize with STIM1 (Figures 2D and S5B). It is well established that overexpression of Orai1 alone into cells often suppresses SOCE responses mediated by endogenous STIM1. This so-called dominant-negative effect (Deng et al., 2009) is likely caused by the impairments of the required coupling stoichiometry between STIM1 and Orai1. This typical effect was also observed in the hOrai1-R83E-E149R construct (Figure 2E), further illustrating that this salt bridge is crucial for Orai1 function. Collectively, this newly identified specific mechanical interaction between the Orai1-N terminus and its loop2 region plays a more critical role during the twist-to-open gating motion than previously estimated (Fahrner et al., 2017).

#### Support from Literature

**Figure 3:**
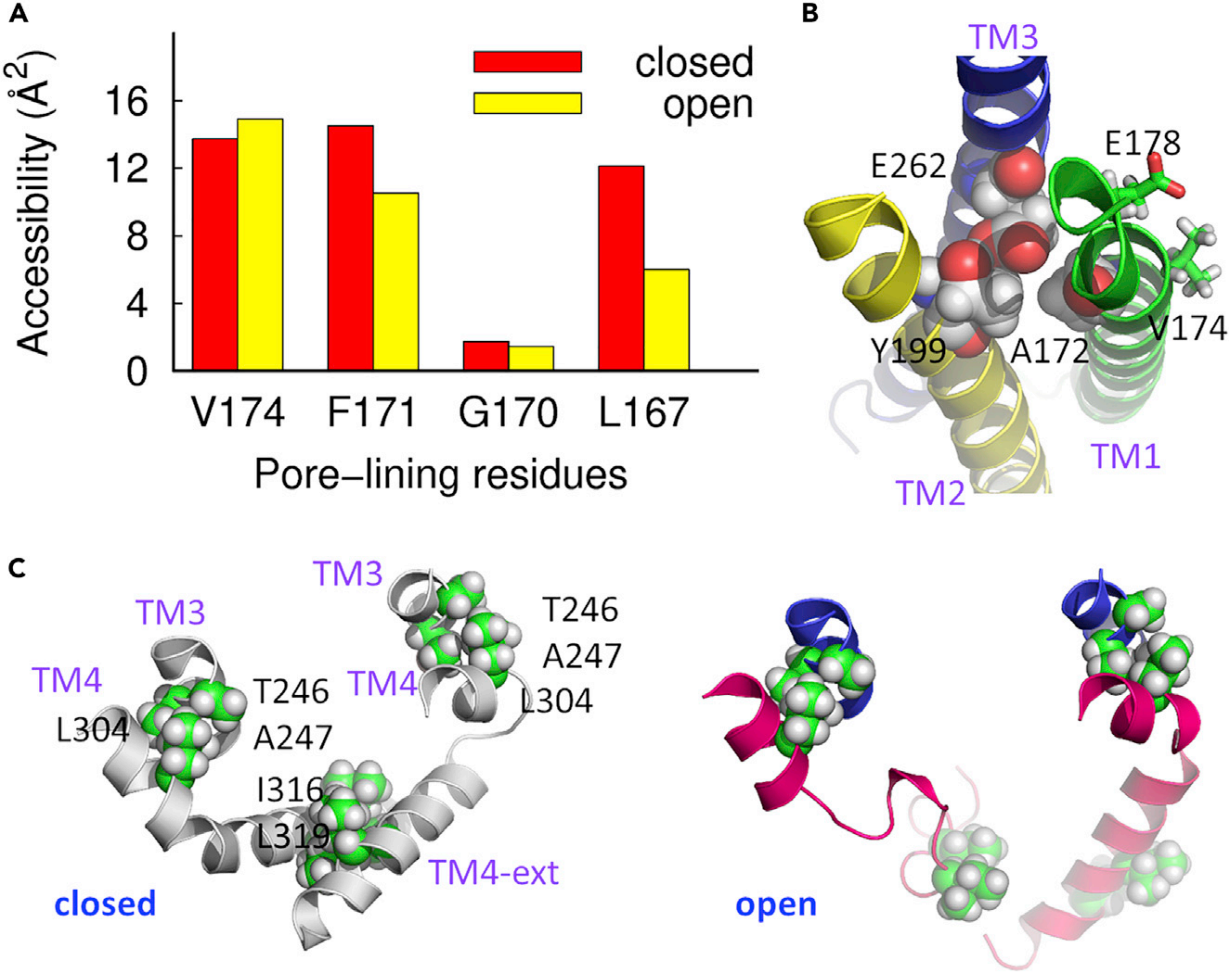
The Open-State Structure Model Reconciles Several Experimental Observations for Orai Gating (A) The change of solvent accessibility of key residues in the pore during gating. (B) The E262 assists to maintain the rigidity of the selectivity filter by forming an atomic interaction network. (C) The well-packed anti-parallel coiled-coil pair on neighboring TM4-ext in the closed state (left panel) moves away upon gating (right panel), whereas the coupling between TM3 and TM4 through the hydrophobic packing between L304 and two consecutive residues T246 and A247 remains intact.

Under STIM1-free conditions, Cd^2+^ ions block the current of the hOrai1-F99C-V102A construct (equivalent to dOrai-F171C-V174A). The current is restored with the presence of STIM1, indicating that the side chain at position 99 was reoriented away from the pore axis during STIM1-triggered gating; in contrast, the hOrai1-G98C mutant (equivalent to dOrai-G170C) shows an increased accessibility at position 98 upon STIM binding (Yamashita et al., 2017). Based on these observations, Yamashita et al. proposed that in hOrai1 there is a counterclockwise rotation of the pore helix by ∼20° when activated by STIM1 (Yamashita et al., 2017). Our putative open-state structure experienced counterclockwise rotation at the extracellular part of the pore (Figure 1B), in which the residues F171 and L167 rearranged to become 25%–50% less exposed on going from the closed state to the putative open state, whereas the residue G170 remains intact (Figure 3A). The conformational changes at the former two positions significantly release the constricting hydrophobic region along the permeation pathway, and therefore facilitate ion transport, whereas the subtle change of the orientation of G170 is enough for better cross-linking, mainly because of its small side chain.

With regard to the residue V102 in hOrai1 (equivalent to V174 in dOrai), the first pore-lining residue in the hydrophobic region, it was proposed that it reoriented upon the binding of STIM1, as suggested by the increased cross-linking in the V102C mutant (Gudlur et al., 2014). The present calculations show that residue V174 is a little more exposed after activation (Figure 3A), which is indeed more favorable for cross-linking.

It was previously proposed that residue E190 on TM3 of hOrai1 (equivalent to E262 in dOrai) regulates ion selectivity (Prakriya and Lewis, 2006, Yamashita et al., 2007), mainly by indirectly maintaining the geometry of the selectivity filter in the central pore (Zhou et al., 2010, Alavizargar et al., 2018). In the closed-state X-ray structure, the residue E262 forms hydrogen bonds with C198 and Y199, with the methyl group on Y199 pointing to TM1 (Hou et al., 2012); in the present putative open-state structure, although the residue C198 is dynamic, the hydrogen bonding between E262 and Y199 remains intact. More importantly, this hydrogen bond locks the orientation of side chain Y199 so that it always points to the periphery of TM1 (Figure 3B). Therefore E190 assists in maintaining the rigidity of the selectivity filter by formation of a key atomic interaction network.

TM4-exts at the cytoplasmic C-terminal domain of Orai1 are the most peripheral segments. This is also the primary STIM1-binding site in hOrai1 (Tirado-Lee et al., 2015, Stathopulos et al., 2013, Zheng et al., 2013, Mcnally et al., 2009, Mcnally et al., 2013, Muik et al., 2008). FRET measurements suggested that the self-associated coiled-coil structure, including residues I316 and L319 (equivalent to L273 and L276 in hOrai1) on adjacent helices, of TM4-ext at the cytoplasmic C-terminal domain of Orai1 experiences conformational change upon STIM1 binding (Tirado-Lee et al., 2015, Mcnally et al., 2013, Navarro-Borelly et al., 2008). In the putative open-state structure, rearrangement of TM4-ext was observed in which the tight packing between the two adjacent TM4-exts is partially broken, and these two residues are no longer paired (Figure 3C). This is accompanied by the hinge bending and swiveling of the segment containing residues L304 V305, S306, and H307 (equivalent to L261, V262, S263, H264, and K265 in hOrai1), the nexus connecting TM4 and TM4-ext (Zhou et al., 2016). However, the tight packing between L261 and two consecutive residues L174 and A175 on TM3 (equivalent to L304, T246, and A247 in dOrai) is almost unimpaired in the putative open-state structure (Figure 3C), which was proposed to be critical for the coupling between TM3 and TM4 (Zhou et al., 2016). Experimentally, mutations L261K, L261D, L174D, or L174K were found to have greatly decreased or eliminated current, although the binding of STIM remains intact (Zhou et al., 2016). This is understandable as the hydrophobic packing is broken, which attenuates the coupling between TM3 and TM4.

### Comparison with Other Constitutively Open-State Structures

A regulatory effect of TM connectivity by substitution of H134 in hOrai1 (equivalent to H206 in dOrai) has been proposed, where the interactions between this amino acid and the neighboring residues determine pore conductance and selectivity (Frischauf et al., 2017). They showed that H134A has locally increased pore size as well as increased flexibility in the basic region, which is in line with what is observed here (Figures 1A and S2). Yeung et al. identified the importance of helix packing, where the helix TM1 in H134S was found to be reoriented with increased pore hydration (Yeung et al., 2018). Hou et al. reported the X-ray structure of dOrai-H206A (Hou et al., 2018). Although there are apparent differences between TM4/TM4-ext, there is a significant dilation of the pore at the N-terminal part along with an observed outward bending of TM1, which is consistent with our putative open-state structure (Figure S3). The observed large-scale reorientation of TM4-ext to insert into the cytosolic side in the dOrai-H206A structure is likely due to crystal packing (Hou et al., 2018), as evidenced by the close contact between two TM4-exts on neighboring constructs in that X-ray structure (Figure S4). A very recent work from Shen and coworkers reported both crystal structure and cryoelectron microscopic (cryo-EM) structures of constitutively open dOrai-P288L (Liu et al., 2019). The outward twisting of TM1 at the basic region was found, which is attributed to the coupling between central pore and the peripheral helices. Similar to that in dOrai-H206A (Hou et al., 2018), TM4 in dOrai-P288L is fully extended, although the cryo-EM density of the TM4-ext segment is invisible (Liu et al., 2019). Notably, the observed aggregation of anions at the cytosolic side of the pore in dOrai-P288L (Liu et al., 2019) experimentally confirmed our predicted anion-assisted cation permeation mode in dOrai-V174A mutant by using molecular modeling (Dong et al., 2014). We want to mention that although different mutant structures share some similarities, they each have unique features (for example, the pore dilation in the dOrai-H206A structure and TM1 twisting at the basic region in the dOrai-P288L structure), indicating that these constitutively open mutants may not well represent the gating of wild-type Orai.

### The Coupling between TM Helices and STIM1-Mediated Orai1 Gating

**Figure 4:**
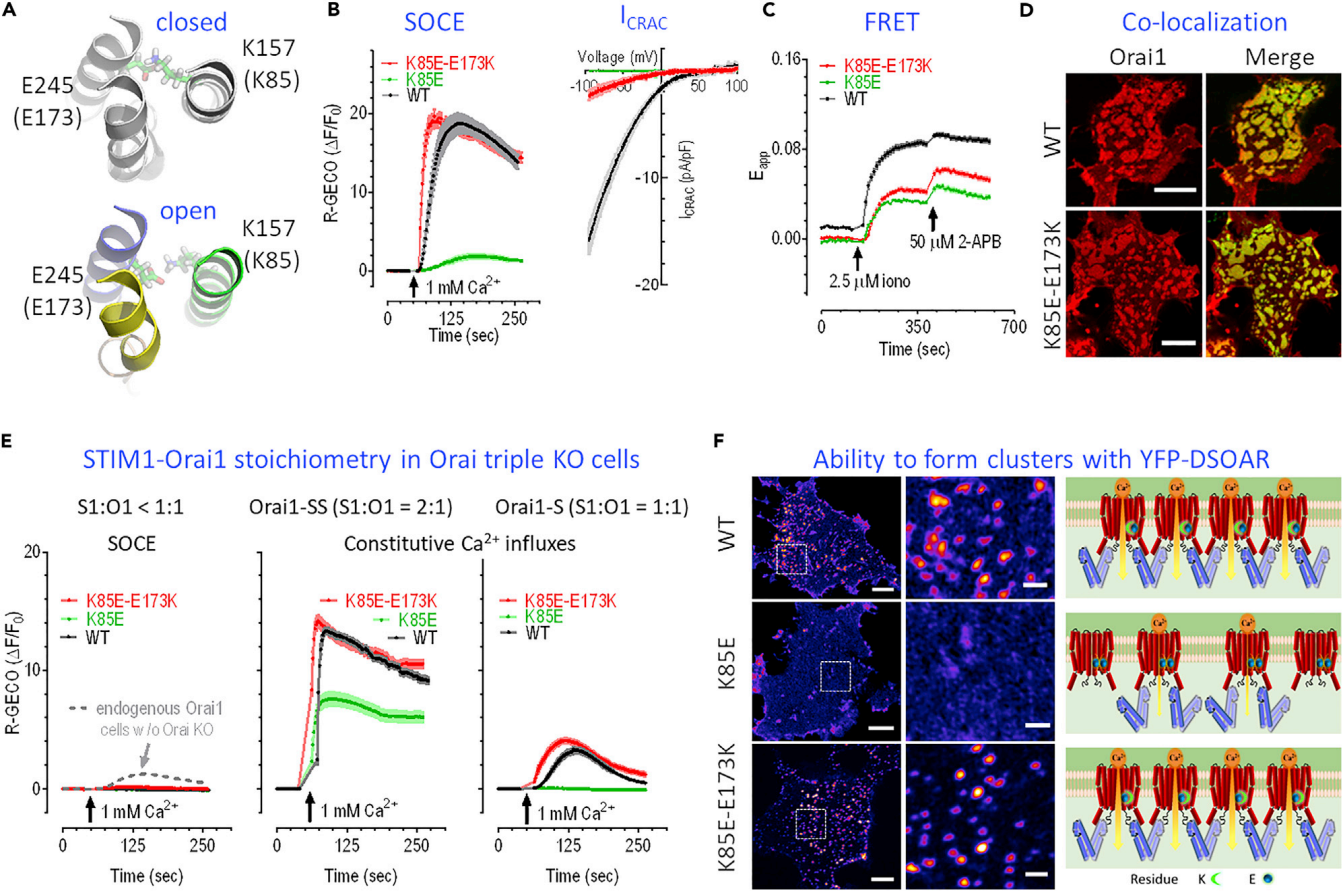
The Mechanical Interplay between K157 and E245 in dOrai Has Implications for Orai-STIM Interaction (A) The salt bridge remains intact during gating. (B–D) In HEK STIM1-YFP cells, the effects of transiently expressed hOrai1-K85E or hOrai1-K85E-E173K mutation were examined. (B) Left, typical SOCE responses; right, mean I-V relationships measured at the peak of whole-cell current (n ≥ 5 for each condition). Mean SOCE: 16.18 ± 0.58 (n = 58) for wild-type [WT], 1.30 ± 0.19 (n = 18) for K85E, and 16.47 ± 0.55 (n = 19) for K85E-E173K. SOCE responses from K85E mutant were significantly lower than those of WT (^∗∗∗^, t test). Please see Figure S5B for typical time courses and statistics of current measurements. (C) FRET signals between STIM1 and hOrai1 before and after the addition of 2.5 μM ionomycin. Ionomycin-induced peak ΔE_app_: 0.028 ± 0.001 (n = 77) for K85E, 0.033 ± 0.001(n = 84) for K85E-E173K. Both are significantly lower than that of WT (0.073 ± 0.002, n = 73) (^∗∗∗^, t test). (D) Confocal imaging results showing typical co-localization of Orai1s with STIM1 after store depletion (scale bar, 10 μm). Please see Figure S5A for complete set of images, and Figure S5B for statistics. (E and F) (E) In Orai1-3 triple KO cells, the effects of K85E or K85E-E173K mutation on SOCE responses mediated by overexpressed Orai1 and endogenous STIM1 (left panel), constitutive Ca^2+^ entry through overexpressed Orai1-SS (middle panel), or Orai1-S (right panel). Mean constitutive Ca^2+^ entry through overexpressed Orai1-SS: WT = 11.17 ± 0.25 (n = 85), K85E = 6.62 ± 0.45 (^∗∗∗^, n = 58), K85E-E173K = 11.74 ± 0.38 (n = 91) (middle panel); mean constitutive Ca^2+^ entry through overexpressed Orai1-S: WT = 1.74 ± 0.13 (n = 75), K85E = 0.02 ± 0.01 (^∗∗∗^, n = 62), K85E-E173K = 2.61 ± 0.16 (^∗∗∗^, n = 89). (t test against control). (F) Airyscan confocal images of YFP-DSOAR1 co-expressed with Orai1 wild-type or its corresponding mutants in STIM1 and STIM2 double KO cells we recently made (Zheng et al., 2018). Left: typical images (scale bar, 5 μm). Middle: magnified detail of the boxed area shown on the left (scale bar, 1 μm). Statistics of cluster densities: 0.28 ± 0.04/μm^2^ for wild-type, 0.04 ± 0.01/μm^2^ for K85E, and 0.31 ± 0.07/μm^2^ for K85E-E173K. Right: hypothetical clustering of wild-type or mutated Orai1 channels by SOAR1 dimers. For (B), (D), (E), and (F) cells were bathed in 0Ca^2+^ solution containing 1 μM TG for 10 min to deplete ER Ca^2+^ store. 1 μM TG was present throughout the recordings. For current measurements, ER Ca^2+^ store was passively depleted by 20 mM BAPTA included in pipette solution. At least three independent repeats were carried out for each experiment.

Our putative open-state structure predicts that the direct coupling between TM1 and TM3 is associated with wild-type dOrai activation. As a conserved mode of action, this coupling was also suggested to be critical for gating in a recently published dOrai-P288L mutant structure (Liu et al., 2019). Previous results in hOrai1-ANSGA mutants indicated that couplings between TM helices are associated with hOrai1 gating (Zhou et al., 2016). Critical for the STIM1-mediated gating (Mcnally et al., 2013, Lis et al., 2010), the K157 residue on TM1 in dOrai (equivalent to K85 in hOrai1) was found to form a salt bridge with E245 on TM3 (equivalent to E173 in hOrai1) from the same subunit in the closed-state structure (Hou et al., 2012). The putative activated dOrai structure shows that the K157-E245 interaction is maintained (Figure 4A), indicating a functional role of this interaction during channel activation. Presumably, the charge reversal mutation at K157 will break the salt bridge and thus impair the coupling between TM1 and TM3, resulting in diminished Ca^2+^ current and weaker coupling between STIM1 and Orai1 (Mcnally et al., 2013, Lis et al., 2010). This was demonstrated here by the hOrai1-K85E construct with significantly decreased Ca^2+^ influx (Figure 4B). The double charge reversal hOrai1-K85E-E173K with re-established salt bridge results in a recovery of SOCE (left panel in Figure 4B), exactly as expected. Even though the K85E-E173K double mutation only partially rescued the diminished current mediated by Orai1-K85E, the I-V relationship of cells expressing hOrai1-K85E-E173K showed inward rectification with very positive reversal potentials (55.3 ± 3.8 mV, n = 16) (right panel in Figures 4B and S5C), signatures for wild-type Orai1 channels. This revealed that the activation of this double mutant is biophysically similar to that of wild-type Orai1 channels. Evidence from both the FRET (Figure 4C) and the co-localization (Figures 4D, S5A, and S5B) analyses indicates that the hOrai1-K85E-E173K mutant exhibits some recovery of the impaired coupling of hOrai1-K85E with STIM1. As the K85E-E173K mutant did not fully rescue the impaired FRET signals caused by K85E mutation, this result indicates that this salt bridge may affect more on intra-Orai1 conformational changes that occur during Orai1 activation. In addition, similar to the wild-type hOrai1 (Prakriya and Lewis, 2015), an excess amount of hOrai1-K85E-E173K has a dominant negative effect on endogenous STIM1, as overexpressed hOrai1-K85E-E173K only induced minimal SOCE in Orai1-3 triple knockout (KO) (Orai KO) cells we recently made (Figure 4E) (Zheng et al., 2018).

To gain further insights into how TM1-TM3 interactions are involved in STIM1-mediated Orai1 activation, we examined whether this interaction is maintained throughout the activation process of hOrai1. It is well established that hOrai1 will be partially activated when the STIM1_336-485_-hOrai1 stoichiometry is 1:1 (denoted as hOrai1-S), and be fully activated with the ratio of 2 (denoted as hOrai1-SS) (Li et al., 2011). We thus generated K85E or K85E-E173K mutations in hOrai1-S or hOrai1-SS concatemers, and examined the effects of mutations on the constitutive Ca^2+^ influxes mediated by these constructs. When expressed in Orai KO cells, the large constitutive Ca^2+^ entry induced by the hOrai1-SS-K85E-E173K mutant is similar to that of hOrai1-SS, with both influxes larger than those mediated by hOrai1-SS-K85E, which is consistent with the results from full-length STIM1 and hOrai1 (Figure 4E, middle panel), showing that TM1-TM3 interactions are crucial for the function of fully activated Orai1 channels. The small constitutive Ca^2+^ influx mediated by partially active hOrai1-S is fully eliminated by K85E mutation, and further K85E-E173K double mutation fully recovers the constitutive Ca^2+^ entry (Figure 4E, right panel). These results clearly demonstrate that the TM1-TM3 interaction is essential for partial activation of Orai1 channels.

hOrai1 cross-linking by a limited amount of the STIM-Orai-activating region (SOAR) was shown to be essential for getting more hOrai1 activated (Zhou et al., 2018, Yuan et al., 2009, Park et al., 2009). Using high-resolution Airyscan confocal imaging, we examined whether TM1-TM3 interactions are also crucial for the newly discovered aspects of Orai1 activation, namely, the cross-linking of hOrai1 channels. We found that the K85E mutation severely impaired the ability of SOAR concatemer-dimers (DSOAR) to cross-link hOrai1 channels, whereas the K85E-E173K mutant regained its ability to be cross-linked by DSOAR with similar amplitude to that of the wild-type hOrai1 (Figure 4F). This observation provides clear evidence showing that the TM1-TM3 interaction within each subunit is also crucial for the cross-linking of hOrai1 channels by DSOAR.

Taken together, our results demonstrated that the K85-E173 salt bridge in hOrai1 between TM1-TM3 is crucial for the STIM1-mediated cross-linking and graded activation of hOrai1 channels, therefore confirming the prediction from our model and illustrating the robustness of the putative open structure in dissecting the mechanisms underlying Orai activation.

## Discussion

The pore-forming TM1 helices are tightly wrapped by peripheral TM2-TM4. During the pore-breathing motion, different conformational changes happen at the N-terminal side of TM1, which are likely associated with two alternating conformations of TM4-ext on neighboring subunits that are tightly packed with each other in the closed state (Hou et al., 2012): the central pore formed by TM1 shows a 6-fold symmetry, whereas the overall dOrai structure adopts a rotational 3-fold symmetry. This symmetry mismatch and local asymmetry is likely to have at least one important consequence: STIM may have various binding modes with Orai, leading to a sequence of conformational changes and therefore different sub-conductance states, which was recently observed by single-channel optical recording (Dynes et al., 2016) and biochemical measurements (Palty et al., 2017). In addition, the pore was found to have graded regulation of current density and ion selectivity by different amounts of STIM1 binding (Li et al., 2011). It is worth noting that AMPA receptor also exhibits a symmetry mismatch (Sobolevsky et al., 2009). In that case, the symmetry transformation between the ligand-binding domain and TM domain was proposed to have implications for the different functions of linkers (Dong and Zhou, 2011).

The primary focus of this work is to identify the gating motion of the wild-type CRAC channel, and a “twist-to-open” mechanism is proposed. This model is different from those proposed based on structural or biochemical measurements of mutants (Yamashita et al., 2017, Frischauf et al., 2017, Hou et al., 2018, Liu et al., 2019). Interestingly, this “twist-to-open” gating mode has been broadly used by other ion channels, such as the G-protein-gated inward rectifier K^+^ (GIRK) channels (Whorton and Mackinnon, 2013), the mechanosensitive channel (MscL) (Perozo et al., 2002, Bocquet et al., 2009), the pentameric ligand-gated ion channels (pLGIC) (Bocquet et al., 2009), and the α7 nAChR (Cheng et al., 2006). A common feature among these channels is the presence of hydrophobic gating in the central pore, which offers a free energy barrier for ion permeation. Although the factors involved in hydrophobic gating, such as pore size regulating the channel conductance, is system dependent (Aryal et al., 2015), seemingly the “twist-to-open” motion identified herein is energetically efficient in disrupting the hydrophobic barrier by introducing a subtle conformational change on the pore, and thereby releasing the inner gate along the ion permeation pathway.

In summary, computational approaches have been employed to derive a putative open-state structure for Orai, and thereby provide a model for its gating mechanism. The present work suggests that activation of the wild-type Orai pore involves a “twist-to-open” mechanism, in which a series of motions lead to the release of the restrained pore without relaxing overall structural integrity. The consistency between new and existing experimental observations and the putative open-state structure, with its computed low free energy barrier for ion permeation and characteristic I-V curve, suggest that the conformational changes to generate the putative open-state structure indeed represent the gating motion for the wild-type Orai pore induced by STIM1 binding. Protein engineering, imaging, and current measurements support the proposed key interactions in the channel and provide further evidence that the TM1-TM3 interaction within each Orai subunit is also crucial for their cross-linking by STIM1. Therefore the proposed activation mechanism inferred from the present computer simulations, and supported by new experiments, provide a working model for activation of CRAC channels under physiological conditions.

### Limitation of the Study

The activation model of Orai pore was proposed with molecular modeling, and obtained validations from wet laboratory experiments and supports from literature. Although all the data presented in this work are consistent with each other, so far, no experimental structure of the wild-type pore in activated state is available to justify the theoretical model. In addition, the detailed mechanism underlying the interaction between Orai pore and its activator STIM1 remains to be defined in future work.

## Methods

All methods can be found in the accompanying Transparent Methods supplemental file.

## Supplemental Information

Document S1. Transparent Methods and Figures S1–S6
