# Supplementary material for: Toward a Model for Activation of Orai Channel
Source: iScience. 2019 Jun 1;16:356–67. doi: 10.1016/j.isci.2019.05.041 (PMC6579751; doi:10.1016/j.isci.2019.05.041)
Supplement: Document S1. Transparent Methods and Figures S1–S6 [file mmc1.pdf]

ISCI, Volume *16*

Supplemental Information

Toward a Model

for Activation of Orai Channel

Hao Dong, Yiming Zhang, Ruiheng Song, Jingjie Xu, Yigao Yuan, Jindou Liu, Jia Li, Sisi Zheng, Tiantian Liu, Benzhuo Lu, Youjun Wang, and Michael L. Klein

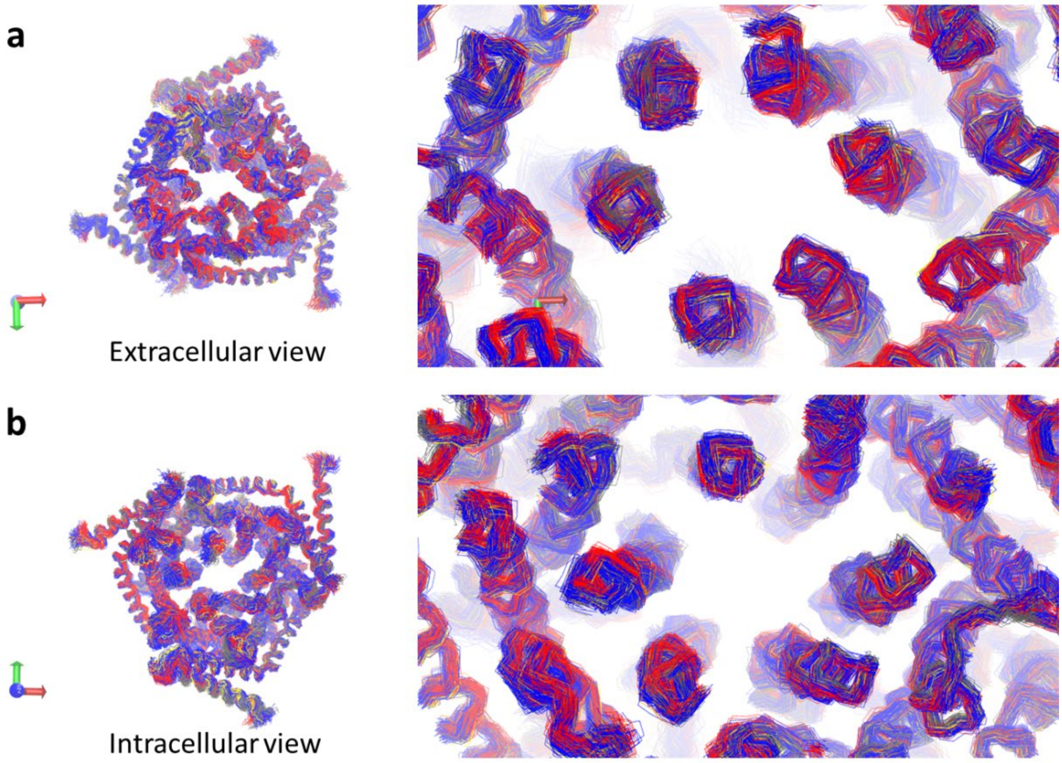

Figure S1. Conformational change of the wild type dOrai pore extracted from clustering analysis of the last 200 ns MD simulations trajectory starting from the closed state structure (4HKR.pdb), related to Figure 1. (a) Extracellular and (b) intracellular views highlight the C- and N-terminal sides of the pore, respectively. Only backbone was shown for clarify. The left panels show the pore, and the right ones are zoomed in to highlight the pore-forming TM1 helices. The K-means algorithm was used for clustering analysis. Among five clusters generated, the major two with the largest number of frames were colored in red and blue, respectively.

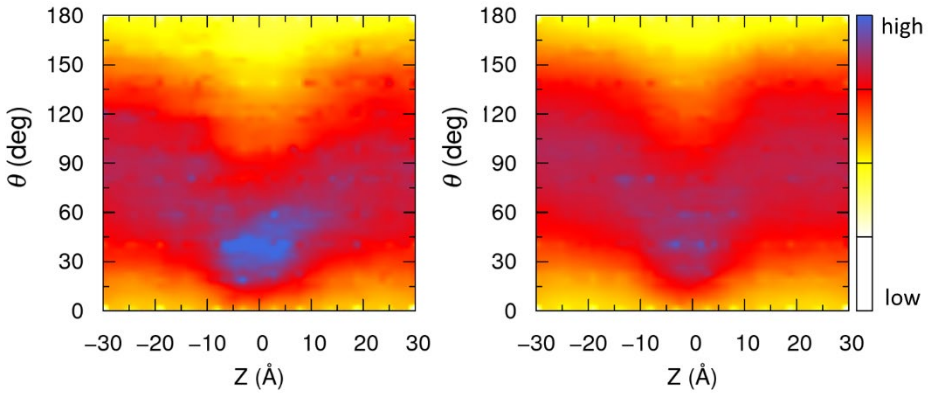

Figure S2. Distribution of θ, the orientation of pore water molecule dipole moments with respect to the pore axis *Z*, in the closed (left) and the putative open (right) constructs of the wild type dOrai channel, related to Figure 1. Waters in the open pore are floppier, as shown by their broader distribution of dipole moment directions.

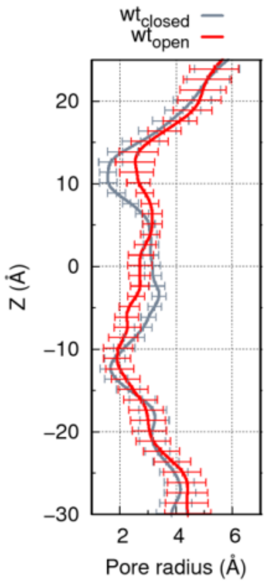

Figure S3. Averaged radii of the pore in different channel constructs obtained from MD simulations, related to Figure 1. The data for the closed and the putative open state of the wild type dOrai are in grey and red, respectively. The error bars represent the standard deviation of each point along the channel. The pore size was calculated with the program Hole (http://www.holeprogram.org).

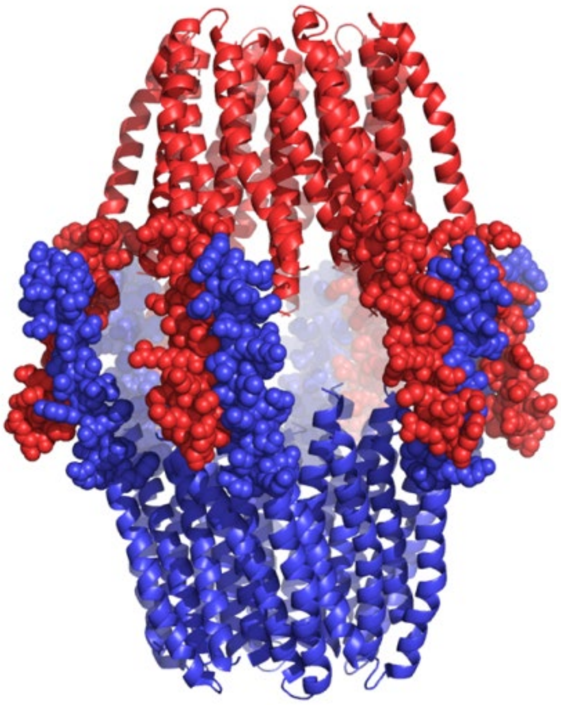

Figure S4. Crystal packing between neighboring constructs in the dOrai-H206A structure (PDB entry: 6BBF), related to Figure 1. Two Orai structures (in red or blue) are shown in cartoon model, while some residues on TM4/TM4-ext (residue ID 301-327) are represented in space-filling mode, showing close contact between them.

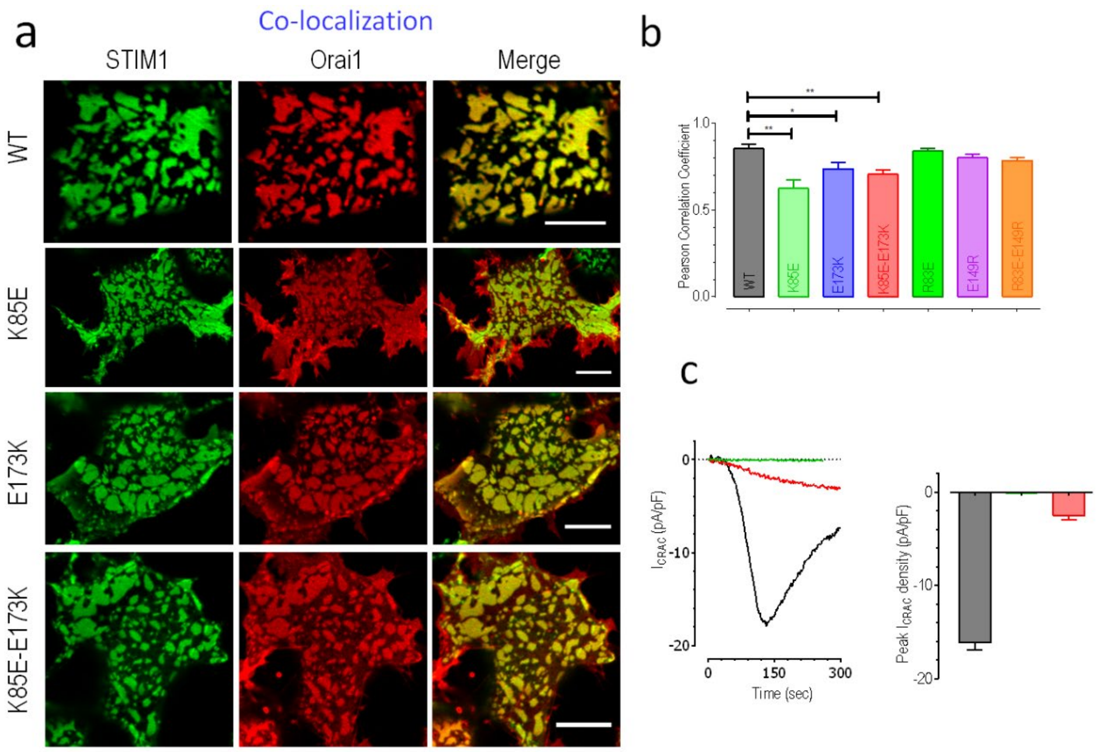

Figure S5. Function and cellular distribution of SOCE proteins in STIM1-YFP cells transiently expressing WT Orai1 or its corresponding mutants, related to Figure 4. a) Typical Confocal images showing the co-localizations between STIM1-YFP and CFP-Orai1 or its corresponding mutants. Cells were pre-treated with 1 μM TG to deplete ER Ca^{2+} stores. (scale bar: 10μm) n=3. b) Quantifications of co-localizations between STIM1 and WT Orai1 or its corresponding mutants (At least 12 cells from 3 independent repeats were examined for each condition). c) Whole cell currents. Top, typical time traces; Bottom, statistics of peak I_{CRAC} amplitudes.

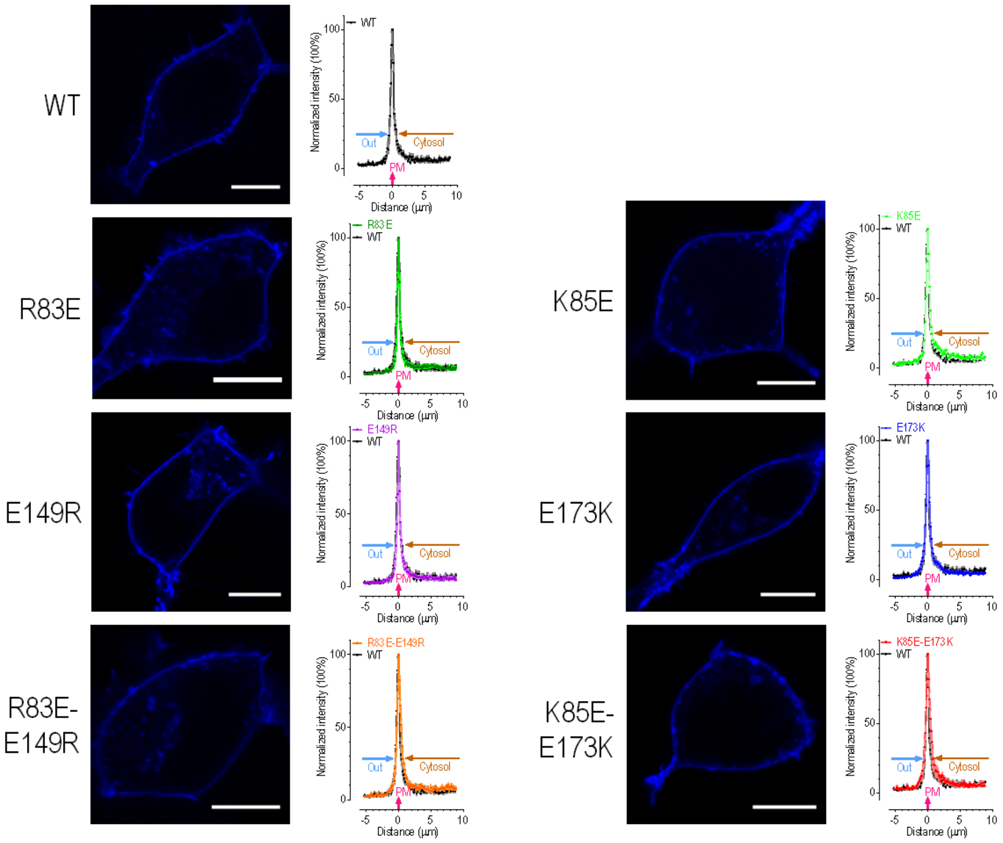

Figure S6. Cellular distribution wild-type CFP-Orai1 (WT) or corresponding mutants transiently expressed in HEK STIM1-YFP cells, related to Figure 4. Left: Typical confocal images; Right: Intensity plots representing the localization of CFP-Orai1 (n=11), K85E (n=11), E173K (n=14), K85E-E173K (n=12), R83E (n=13), E149R (n=17), and R83E-E149R (n=14) across the cell edges (Cells were randomly chosen from three independent from repeats).

Transparent Methods

(1) Normal mode analysis (NMA)

The x-ray structure of dOrai in the closed state (PDB code: 4HKR) (1) was taken as the initial structure to explore the putative open state structure. Only two subunits among six were reported in the x-ray structure, therefore the hexameric structure of dOrai was built by using an appropriate symmetry operation. Normal mode analysis was carried out with the elNémo program (2). Elastic connections were made between each Cα atom and its neighbors within a 12-Å cutoff. For the selected direction of motion, an amplitude of 1 Å was used to generate the initial configuration of the transmembrane (TM) helices in the putative open state structure. Then the missing side-chains and loops connecting TM helices were added. The whole protein was energy minimized with harmonic restraints on protein Cα atoms of the TM helices to remove bad contacts and structural distortions.

(2) Molecular dynamics (MD) simulations

The putative open state structure of dOrai was embedded in a fully hydrated 1-palmitoyl-2-oleoyl-sn-glycero-3-phosphocholine (POPC) bilayer. The membrane bilayer system was solvated by 27,598 water molecules, 74 sodium ions, and 65 chloride ions, where the extra 9 cations served to neutralize the whole system. The total number of atoms in the periodically replicated simulation box size of size 125×125×105 $Å^3$, is 139,504.

The system was energy minimized, with harmonic restraints on the protein Cα atoms, to remove bad contacts and structural distortion. The simulation system was then equilibrated, with harmonic position restraints applied to the heavy atoms of the protein backbone, in the constant temperature and pressure (NPT) ensemble. The force constant for constraint started at 100 kcal/mol, and was gradually decreased to zero during a 200 ns MD trajectory. For each system studied, three independent MD simulations were carried out, data from each of them were accumulated for 600 ns.

The MD simulations were carried out using the CUDA-accelerated NAMD program version 2.12 (11). The Charmm36 force field parameters (12) and the TIP3P water model were used (13). Periodic boundary conditions were applied and the particle mesh Ewald method was used to treat long-range electrostatic interactions.

(3) Potential of mean force (PMF) calculations

To characterize the energy cost for cation permeation through the central pore, the free energy profiles for the translocation of a single Na^+ ion between the

extracellular and intracellular sides of the system were calculated by employing the adaptive biasing force (ABF) simulation methodology (14).

The needed reaction coordinate (RC) was defined as the position of the ion along the z axis (parallel to the membrane normal), which is in the range of −56 Å ~ +36 Å along the z direction where z=0 is the center of the lipid bilayer. To fully cover the reaction coordinate, 23 evenly distributed windows were used, with the width of 4 Å for each window. Quasi-equilibrium trajectories generated by ABF (14) were used to determine the PMF by fully sampling the configuration space in each window. A force constant of 100 kcal/mol was imposed at the boundary of each window, and the width of bin size of 0.1 Å was used. Simulations in each window were continued until the convergence of PMF was achieved. The total accumulated simulation time of trajectories used for the PMF calculations is 3.45 μs for the putative open state of the wild type pore of dOrai.

(4) Reconstructing 2-dimensional (2D) PMF from 1-dimensional (1D) PMF

Though the ABF simulations can efficiently explore the free energy surface with enhanced sampling techniques, a huge amount of time (2 to 3 μs) is needed to get the 1D PMF profile in the present case, and the sampling needed in 2D space is almost computationally prohibited. Therefore, we estimated the 2D free energy landscape for cation permeation along the central pore from the abovementioned 1D PMF data. The second RC, RC2, was defined as the motion of the cation in the x-y plane (i.e., perpendicular to the membrane normal), which is orthogonal to the first RC, the permeation of the cation along the z-axis. However, the bias acting on the collective variables distorts the probability distribution of the other variables. Therefore, reweighting the distribution along the reaction coordinate in the 1D PMF profile is a prerequisite for reconstructing the 2D free energy landscape. Inspired by the work of Parrinello *et al.* (15), where an efficient protocol was proposed to reweight the biased distributions obtained from metadynamics calculations, we established the relation between the biased probability distribution and the Boltzmann one based on ABF simulations, as described below.

For the fully converged sampling along RC1, the calculated PMF was canceled out by an equal and opposite biasing force. As shown in equation (1), the biased distribution, P_{bias}, is affected by the applied biasing force, where $\beta=1/k_B T$, k_B is Boltzmann's constant, T is the temperature, $N_{i,j}$ is the population in the window when RC1=i and RC2=j, and $V_{i,j}$ is the biasing potential energy applied in this window. By adding the additional RC2 and dividing the 2D free energy surface into small bins, the distribution and the corresponding histogram in each bin could was counted. Then, by extracting the bias from the distribution in each bin, we estimated the unbiased distribution, P_{real}, with equation (2). The final 2D-PMF was obtained by combining

all the bins along RC1, where the boundaries of each bin were smoothly connected.

$$P_{bias} = \frac{N_{i,\ j}}{\sum_{i,\ j} N_{i,\ j}} = \frac{Exp(-\beta E_{i,j}) * Exp(-\beta V_{i,j})}{\sum_{i,\ j} Exp(-\beta E_{i,j}) * Exp(-\beta V_{i,j})} \quad (1)$$

$$P_{real} = \frac{Exp(-\beta E_{i,j})}{\sum_{i,\ j} Exp(-\beta E_{i,j})} = \frac{N_{i,\ j} * Exp(\beta V_{i,j})}{\sum_{i,\ j} N_{i,\ j} * Exp(\beta V_{i,j})} \quad (2)$$

(5) Calculation of the current-voltage (I-V) relationship

5.1 The post-processing protocol for modeling the ion current

MD simulations focus on the dynamics and interactions of every particle in a nanoscale system with femtosecond resolution. Thus, it is computationally demanding to simulate typical ion permeation processes (nanoseconds to milliseconds timescale) in a channel. By contrast, a continuum model, such as the Poisson-Nernst-Planck (PNP) model, ignores certain particle interaction details, but achieves high efficiency in simulating the transport phenomenon. Therefore, one approach is to combine MD and PNP methodologies in order to model the ion current. Generally speaking, the idea of this combined approach is to select representative configurations of the pore that are sampled from a large-scale atomistic MD simulation trajectory, and then apply the PNP model to each of the representative structures. Averaging the calculated data over an ensemble of MD trajectory configurations is likely to minimize errors coming from the sensitivity of the calculated current to the chosen configuration of the channel. In the present work, 10 snapshots taken from the MD simulations of each construct were used for the following continuum model calculations. For each snapshot, the currents at 200 mV, 100 mV, 50 mV, 20 mV, 10 mV, 0 mV, -10 mV, -20 mV, -50 mV, -100 mV and -200 mV were calculated, respectively. For each voltage, the calculated currents from different structures were averaged before plotting the final I-V relationship. Further details about the PNP model and its implementation in this work are given below.

5.2 The PNP model

The PNP model is the most commonly used continuum model to simulate the macroscopic transportation properties of ion channels (16-20). The model couples a set of drift-diffusion equations (including a Fickian diffusion term and a drift term accounting for the effects of the electrostatic field) and a Poisson equation describing the electrostatic potential field determined by the fixed charges in the channel protein and the mobile ion concentrations. In this work, the I-V curves were obtained through solving the steady-state PNP equations:

$$\nabla \cdot D_i(\nabla c_i + \beta q_i c_i \nabla \phi) = 0 \quad (3)$$

$$\nabla \cdot \varepsilon \nabla \phi = -\sum_i q_i c_i - \rho^f \quad (4)$$

where β=1/k_BT, k_B is the Boltzmann's constant, T is the temperature, C_i denotes the concentration distribution function for the ith ion species with a diffusion coefficient D_i, and charge q_i on each particle, φ denotes the electrostatic potential, ε the dielectric constant, and ρ^f denotes the fixed atomic charges distribution in membrane protein.

Given a membrane voltage (treated as boundary conditions in the PNP model), the ionic concentration distribution functions and electrostatic potential are obtained through solution of the PNP equations. Then, the ionic current in a channel can be calculated by integrating the flux density on a cross-section, S of the channel:

$$I = -\sum_i q_i \int_S D_i \left(\frac{\partial c_i}{\partial z} + \beta q_i c_i \frac{\partial \phi}{\partial z}\right) dS \quad (5)$$

5.3 The system setup

We use the finite element method (FEM) to solve the PNP model. A qualified tetrahedral mesh is crucial for the numerical solution. Four steps are needed to generate a high-quality tetrahedral mesh for a membrane channel protein: First, our previously developed mesh generation program TMSmesh is used to read the PQR file (containing the atomic charges and radii information) and triangulate a Gaussian surface for the protein molecule (21, 22). Next, the quality of the triangular surface mesh is improved; and then TetGen (23) is used to generate a tetrahedral volume mesh based on the surface mesh. Finally, a membrane region is marked in the computational domain. Mesh generation is a challenging task due to the complicated and irregular molecular geometry of the ion channel.

A dielectric constant of ε=78 was chosen for the solvent regions, and ε=2 for both the membrane and the protein regions. Since the channel pore serves as a confining space for the mobile ions, a position-dependent diffusion coefficient is employed:

$$D(z) = \begin{cases} D_{\text{bulk}} & z \text{ in the bulk region} \\ D_{\text{chan}} + (D_{\text{chan}} - D_{\text{bulk}}) f(z) & z \text{ in the buffer region} \\ D_{\text{chan}} & z \text{ in the channel region} \end{cases} \quad (6)$$

where the function $f(z)$ is given by

$$f(z) = n \cdot \left(\frac{z - z_{\text{chan}}}{z_{\text{bulk}} - z_{\text{chan}}}\right)^{n+1} - (n+1) \cdot \left(\frac{z - z_{\text{chan}}}{z_{\text{bulk}} - z_{\text{chan}}}\right)^{n} \quad (7)$$

and n is set to 7 in the present work, D_{bulk} and D_{chan} are the diffusion coefficients in the bulk and channel regions, respectively. Z_{chan} is the boundary value of the channel region on the z axis, and Z_{bulk} is the boundary value of bulk region on the z axis.

5.4 The finite element method (FEM)

The PNP equations are solved in a decoupled and iterative approach: First, we solve for the potential in the Poisson equation with the FEM for given initial ion concentrations, next, solve each of the NP equations with the FEM to obtain new ion concentrations, and then iteratively repeat these two procedures until convergence. To make the iterations between the NP and the Poisson equations converge, a relaxation scheme is needed at each step for solution updating:

$$\tilde{\phi}^n = \alpha\phi^{n-1} + (1-\alpha)\phi^n$$
$$\tilde{c}_i^n = \alpha c_i^{n-1} + (1-\alpha)c_i^n \quad (8)$$

where $0<\alpha<1$ is the relaxation parameter. If the difference between φ^n and φ^{n-1} is smaller than the assigned tolerance, the iteration is stopped. In our simulations, a preconditioned generalized minimal residual method (GMRES) is often used for solving the linear system generated in the FEM, which improves the efficiency and robustness of the algorithm.

(6) DNA constructs, cell culture, and transfection

All Orai1 sequences containing the following mutations, K85E / E173K / K85E-E173K or R83E / E149R / R83E-E149R, were obtained from CFP-Orai1 (26) using overlap PCR, and then sub-cloned into the pECFP-C1 vector using XhoI and BamHI sites. To generate untagged wild-type or mutated Orai1, the sequences were amplified from the corresponding CFP-Orai1 constructs with primers containing BamHI and EcoRI sites, and sub-cloned into the pCDNA3.1(+) vector. The Orai1-SS sequence was amplified from Orai1–SS–eGFP (27), and inserted into pECFP-C1. To generate CFP-Orai1-SS mutants, Orai1 sequences containing desired mutations were amplified from the corresponding CFP-Orai1 constructs with primers containing XhoI and KpnI to replace the original wild-type Orai1 sequence in the CFP-Orai1-SS plasmid. CFP-Orai1-S constructs were generated by digesting out the first S fragment with EcoRI from Orai1-SS, and then ligating the resulting larger product with T4 ligase.

Cell culture: wild-type Human embryonic kidney 293 (HEK wt) cells were maintained in regular DMEM (HyClone) supplemented with 10% FBS (Cleson Scientific), penicillin and streptomycin (Thermo Scientific) at 37°C with 5% CO_2 as previously described (28). HEK STIM1-YFP cells were maintained in the above-mentioned medium supplemented with 2 μg/ml puromycin (Invitrogen) (28).

Transfections: All transfections were performed by electroporation using a voltage step (180V, 25ms), OPTI-MEM medium, 4mm cuvettes (Molecular Bio-Products), and the Bio-Rad Gene Pulser Xcell system as previously described (29).

(7) Ca^{2+} and FRET imaging

Both FRET and Ca^{2+} imaging was conducted at room temperature using a ZEISS oberserver-Z1 microscope equipped with standard Semrock filters, controlled with the Zen software using similar protocols as described previously (28). The imaging solution contained (mM): 107 NaCl, 7.2 KCl, 1.2 $MgCl_2$, 11.5 glucose, and 20 HEPES-NaOH (pH 7.2). For single-cell intracellular cytosolic Ca^{2+} measurements, red fluorescence from cells contransfected with a red Ca^{2+} indicator, R-GECO1.2 (30), were acquired every 2 s using a TxRed-A-Basic-000 filter set. The changes in intracellular Ca^{2+} levels are presented as changes in R-GECO fluorescence ($\Delta F/F_0$). For FRET measurements, standard Semorock CFP YFP filters, and $FRET_{raw}$ (CFP_{Ex}/YFP_{Em}) were used for image acquisition (F_{CFP}, F_{YFP}, and F_{raw}, respectively), every 10 s. System calibrations and off-line analysis were performed with previously described protocols (31). Representative traces from at least three independent experiments are shown as mean ± s.e.m.

(8) Regular and Airy scan confocal imaging

CFP and YFP fluorescent images were acquired at room temperature using a Zen software-controlled ZEISS LSM 880 confocal system equipped with 458, 488, 514nm argon-ion laser, GaAsP-PMT detector. For co-localization experiments, CFP fluorescence (465-520nm) excited by 458nm laser, YFP fluorescence (520-620nm) excited by 514 nm laser were collected with regular PMT detectors using a scanning mode of "switch track every line". There is no visible bleed through between CFP and YFP channels with the current setup. For high resolution Airyscan imaging, the above YFP signals were collected with an array of GaAsP-PMT detectors following the manufacture's protocols. Cluster analysis was done with ImageJ software using previously published protocols (28). Images shown were typical of at least three independent experiments. Error bars denote mean ± s.e.m.

(9) Electrophysiology

Whole cell currents from HEK293 YFP-STIM1 stable cells transiently expressing CFP-Orai1-K85E-E173K, were measured with conventional whole-cell recordings (31). The pipette solution contained (mM): 135 Cs-aspartate, 8 $MgCl_2$, 5 BAPTA, and 10 HEPES (pH 7.2). The bath solution contained (mM): 130 NaCl, 4.5 KCl, 20 $CaCl_2$, 10 TEA-Cl, 10 D-glucose, and 5 HEPES (pH 7.4). A 10-mV junction potential compensation was applied to compensate the liquid junction potential. Currents were obtained with a sampling rate of 10 kHz and filtered at 2.3 kHz (four-pole Bessell). Current responses to sweeps delivered every 2 s were collected with a Patchmaster controlled HEKA EPC 10 USB double patch amplifier (HEKA Elektronik). The resulting data were further offline low-pass filtered at 500 Hz and analyzed with

HEKA Fitmaster and Matlab 2014b software. Results from at least six cells were collected and averaged.

1. Hou X, Pedi L, Diver MM, & Long SB (2012) Crystal structure of the calcium release-activated calcium channel Orai. *Science* 338(6112):1308-1313.
2. Suhre K & Sanejouand Y-H (2004) ElNemo: a normal mode web server for protein movement analysis and the generation of templates for molecular replacement. *Nucleic Acids Res* 32(suppl_2):W610-W614.
3. Yamashita M*, et al.* (2017) STIM1 activates CRAC channels through rotation of the pore helix to open a hydrophobic gate. *Nat Commun* 8:14512.
4. McNally BA, Somasundaram A, Yamashita M, & Prakriya M (2012) Gated regulation of CRAC channel ion selectivity by STIM1. *Nature* 482(7384):241-245.
5. Dong H, Fiorin G, Carnevale V, Treptow W, & Klein ML (2013) Pore waters regulate ion permeation in a calcium release-activated calcium channel. *Proc Natl Acad Sci USA* 110(43):17332-17337.
6. Dong H, Klein ML, & Fiorin G (2014) Counterion-assisted cation transport in a biological calcium channel. *J Phys Chem B* 118(32):9668-9676.
7. Dai J & Zhou HX (2014) General rules for the arrangements and gating motions of pore-lining helices in homomeric ion channels. *Nat Commun* 5:4641.
8. Zhang SL*, et al.* (2011) Mutations in Orai1 transmembrane segment 1 cause STIM1-independent activation of Orai1 channels at glycine 98 and channel closure at arginine 91. *Proc Natl Acad Sci USA* 108(43):17838-17843.
9. Frischauf I*, et al.* (2017) Transmembrane helix connectivity in Orai1 controls two gates for calcium-dependent transcription. *Sci Signal* 10(507):eaao0358.
10. Palty R, Stanley C, & Isacoff EY (2015) Critical role for Orai1 C-terminal domain and TM4 in CRAC channel gating. *Cell Res* 25(8):963-980.
11. Phillips JC*, et al.* (2005) Scalable molecular dynamics with NAMD. *J Comput Chem* 26(16):1781-1802.
12. Best RB*, et al.* (2012) Optimization of the additive CHARMM all-atom protein force field targeting improved sampling of the backbone ϕ, ψ and side-chain $\chi 1$ and $\chi 2$ dihedral angles. *J Chem Theory Comput* 8(9):3257-3273.
13. Jorgensen WL, Chandrasekhar J, Madura JD, Impey RW, & Klein ML (1983) Comparison of simple potential functions for simulating liquid water. *J Chem Phys* 79(2):926-935.
14. Darve E, Rodríguez-Gómez D, & Pohorille A (2008) Adaptive biasing force method for scalar and vector free energy calculations. *J Chem Phys* 128(14):144120.
15. Bonomi M, Barducci A, & Parrinello M (2009) Reconstructing the equilibrium Boltzmann distribution from well-tempered metadynamics. *J Comput Chem* 30(11):1615-1621.
16. Chen D, Lear J, & Eisenberg B (1997) Permeation through an open channel: Poisson-Nernst-Planck theory of a synthetic ionic channel. *Biophys J* 72(1):97-116.
17. Corry B, Kuyucak S, & Chung S-H (2000) Tests of continuum theories as models of ion channels. II. Poisson–Nernst–Planck theory versus Brownian dynamics. *Biophys J* 78(5):2364-2381.

18. Bolintineanu DS, Sayyed-Ahmad A, Davis HT, & Kaznessis YN (2009) Poisson-Nernst-Planck models of nonequilibrium ion electrodiffusion through a protegrin transmembrane pore. *PLoS Comput Biol* 5(1):e1000277.
19. Tu B, *et al.* (2013) A parallel finite element simulator for ion transport through three-dimensional ion channel systems. *J Comput Chem* 34(24):2065-2078.
20. Liu X & Lu B (2017) Incorporating Born solvation energy into the three-dimensional Poisson-Nernst-Planck model to study ion selectivity in KcsA K+ channels. *Phys Rev E* 96(6):062416.
21. Chen M & Lu B (2010) TMSmesh: A robust method for molecular surface mesh generation using a trace technique. *J Chem Theory Comput* 7(1):203-212.
22. Liu T, Bai S, Tu B, Chen M, & Lu B (2015) Membrane-Channel Protein System Mesh Construction for Finite Element Simulations. *Mol Based Math Biol* 3(1).
23. Hang S (2005) A Quality Tetrahedral Mesh Generator and a 3 D Delaunay Triangulator.
24. Sansom MS & Weinstein H (2000) Hinges, swivels and switches: the role of prolines in signalling via transmembrane alpha-helices. *Trends Pharmacol Sci* 21(11):445-451.
25. Cordes FS, Bright JN, & Sansom MS (2002) Proline-induced distortions of transmembrane helices. *J Mol Biol* 323(5):951-960.
26. DeHaven WI, Smyth JT, Boyles RR, & Putney JW (2007) Calcium inhibition and calcium potentiation of Orai1, Orai2, and Orai3 calcium release-activated calcium channels. *J Biol Chem* 282(24):17548-17556.
27. Li Z, *et al.* (2011) Graded activation of CRAC channel by binding of different numbers of STIM1 to Orai1 subunits. *Cell Res* 21(2):305-315.
28. Wei M, *et al.* (2016) Molecular mechanisms underlying inhibition of STIM1-Orai1-mediated Ca 2+ entry induced by 2-aminoethoxydiphenyl borate. *Pflugers Arch* 468(11-12):2061-2074.
29. Hendron E, *et al.* (2014) Potent functional uncoupling between STIM1 and Orai1 by dimeric 2-aminodiphenyl borinate analogs. *Cell Cal* 56(6):482-492.
30. Pathak GP, Vrana JD, & Tucker CL (2013) Optogenetic control of cell function using engineered photoreceptors. *Biol Cell* 105(2):59-72.
31. Ma G, *et al.* (2015) Inside-out Ca 2+ signalling prompted by STIM1 conformational switch. *Nat Commun* 6:7826.
